# Examining infantile facial features and their influence on caretaking behaviors in free-ranging Japanese macaques (*Macaca fuscata*)

**DOI:** 10.1371/journal.pone.0302412

**Published:** 2024-06-20

**Authors:** Toshiki Minami, Takeshi Furuichi

**Affiliations:** 1 Graduate School of Education, Kyoto University, Kyoto, Kyoto, Japan; 2 Wildlife Research Center, Kyoto University, Inuyama, Aichi, Japan; Children’s Hospital Affiliated of Zhengzhou University: Zhengzhou Children’s Hospital, CHINA

## Abstract

Facial features of immature individuals play a pivotal role in eliciting caretaking behaviors in humans. It has been posited that non-human animals share particular infantile facial features with humans, which can elicit caregivers’ attention and caretaking behaviors. Nevertheless, the empirical examination of this hypothesis is extremely limited. In this study, we investigated infantile facial features in Japanese macaques (*Macaca fuscata*), their developmental processes, and their correlation with caretaking and infant behaviors, based on 470 facial photographs from one free-ranging group. We measured the size of facial parts and evaluated these features using non-contact procedures with the animals. The results indicated that, although some partial species differences were observed, the infantile facial features in Japanese macaques were broadly consistent with those previously observed in humans and great apes. Furthermore, half of the infant subjects displayed non-linear developmental trajectories of infantile faces, similar to those suggested in humans. However, unlike previous studies in humans, infantile faces were not significantly associated with maternal or non-maternal caretaking behaviors, nor were their developmental changes correlated with infant behavioral development. These findings indicate that while many aspects of infantile facial features are shared among particular primates, humans may have evolved a uniquely elevated preference for selecting such features among the primate lineage.

## Introduction

Research investigating the characteristics and evolution of human caretaking behaviors compared to those observed in non-human primates is increasingly significant. Primates, including humans, exhibit extended periods of immaturity [1], and care received during this period profoundly influences the survival and growth of juveniles [2−5]. Thus, understanding the factors promoting primate caretaking behaviors can support their sound development. Extensive research has revealed that human caretaking behaviors exhibit unique traits among primates, such as cooperative breeding, prolonged immaturity, and shorter birth intervals [6]. Therefore, comparative studies with non-human primates are essential for understanding the characteristics and evolution of human caretaking behaviors and for exploring the factors that promote such behavior in humans. Such research holds meaningful implications for establishing a caretaking environment appropriate for human beings and achieving the reciprocal well-being of both children and caregivers.

In humans, one of the key triggers for caretaking behaviors is the physical features of immature individuals [7]. Lorenz [8] proposed that infants possess distinct physical features that stimulate their care. While his suggestion encompassed overall morphological characteristics, subsequent studies have highlighted specific facial features, such as a greater facial width relative to head length (i.e., a rounded head), a longer forehead relative to the facial length (i.e., low-set eyes), larger eyes relative to the facial width, and a smaller nose and mouth relative to the facial size [9]. Experimentally enhancing these features when evaluating infant facial stimuli can increase infant attractiveness and capture and retain the observer’s attention [9−17]. Furthermore, watching infant faces can activate neural reward and motivational systems [18] and stimulate caretaking behavior in recipients [9]. Although studies on the links between facial features and caretaking behaviors are restricted, Langlois et al. [19] reported that neonates rated as more attractive by non-caregivers experienced more intimate interactions with their caregiving mothers. These findings suggest that the facial appearance of human infants can promote positive responses from caregivers and potentially elicit tangible caretaking behaviors.

Contrary to our intuition, the prominence of these facial features may not always be more pronounced in less mature infants. For example, when compared to preterm infants of the same age, full-term infants have more emphasized facial features such as forehead length, facial width, and eye width [20]. Furthermore, the development of facial cuteness in infants follows a hump-shaped trajectory with age, peaking between 6 and 11 months of age, rather than immediately postpartum [16, 21–23]. This trend is intriguing, given that newborns are more vulnerable [21]. As a potential explanation for this non-linear developmental change, Negayama [16] proposed a link to infant mobility. The peak age of human infantile facial attractiveness coincides with the period when they begin exploring their environment independent of their caregivers, thereby increasing the likelihood of encountering potential dangers. The most attractive faces at this developmental stage could more strongly capture the attention of caregivers and help mitigate heightened danger. Although it is necessary to examine this hypothesis empirically, the development of infantile features may also have an evolutionary origin that captures elevated attention from caregivers.

Lorenz [8] proposed that infantile morphologies and the increased attention paid to them are widespread across various animal species, a long-acknowledged perspective. However, empirical investigations on non-human animals have only recently begun. For instance, Kawaguchi et al. [24] remains the only study to examine the interspecific similarities and differences in infantile facial features among primates. They compared the facial morphologies of great apes, including humans, and revealed the following conserved facial features in infants: expanded facial width (round and short faces), a prolonged forehead (eyes positioned lower on the face), enlarged eyes, and an inverted triangular configuration. In contrast, the smaller nose and mouth, which are typical characteristics of human infants, were not shared among the great apes as infantile features [24]. To advance our understanding of cross-species similarities and variations in infantile facial features, an examination across species beyond the great apes is necessary.

Research is also underway to explore how non-human primates react to the faces of conspecific infants. By presenting infant and adult images, preference for infant faces has been documented in chimpanzees (*Pan troglodytes*), Barbary macaques (*Macaca sylvanus*), and rhesus macaques (*Macaca mulatta*) [25−27]. However, their reactions were likely to be motivated by infantile facial color rather than facial shape. Additionally, bonobos (*Pan paniscus*) and Japanese macaques (*Macaca fuscata*) did not exhibit a significant preference for infant faces [28, 29]. These investigations suggest that non-human primates are less attracted to infantile faces, whereas humans strongly prefer infantile facial morphology. However, it is unclear whether infantile faces are linked to the recipient’s motivation for caretaking, which can directly contribute to infant survival and development. To test Lorenz’s [8] hypothesis in primates, a novel study on the direct association between infant facial appearance and caretaking behavior is essential.

Whether the hump-shaped development of infantile faces observed in humans is also present in non-human primates remains uncertain. In the only relevant study, Sanefuji et al. [23] presented images of infant chimpanzees, rabbits (*Oryctolagus cuniculus*), dogs (*Canis familiaris*), and cats (*Felis syvestris catus*) to college students. The findings revealed that the most attractive faces across all species were not those of neonates, but rather of infants several months old. Humans perceive the attractiveness of heterospecific faces using the same criteria as they do for conspecific infants [30, 31]. Hence, if infantile facial features are shared across animal taxa [8], this suggests the potential existence of non-linear development of infantile faces in non-human species. Additionally, similar to humans, non-human primate infants would face escalated risks of injury if they began independent exploratory behaviors unattended. Therefore, if infantile faces indeed facilitate caretaking behaviors, non-human infants might benefit from having pronounced facial features during this critical period of their development [16].

To further our understanding of the associations between infantile facial features and caretaking behaviors and to elucidate the applicability of Lorenz’s hypothesis [8] in non-human primates, this study investigated the following four questions using Japanese macaques, one of the non-ape primates. (1) Do Japanese macaques exhibit facial features similar to those of great apes, including humans? [8] (2) Is there a correlation between the physical proportions of infantile faces and caretaking behaviors among infant Japanese macaques? [8] (3) Do the facial proportions of infant Japanese macaques show a hump-shaped development, akin to human infants? [16, 21, 22, 23] (4) Is there a clear association between pronounced infantile faces and the initiation of exploratory behavior in Japanese macaque infants? [16] To answer these questions, we collected a number of facial photographs from nearly all individuals within a single troop of Japanese macaques, quantitatively evaluated their facial features, and examined the development of infantile faces and their potential associations with behavioral patterns.

In this study, we chose a free-ranging troop of Japanese macaques as the study target. The Japanese macaque is a primate species endemic to Japan belonging to the Cercopithecidae. They are considered infants for less than one year after birth, attain sexual maturity at around five years of age, and typically die by the age of approximately 30 years old in our study troop [32]. Free-ranging Japanese macaques met the objectives of this study for the following four reasons. First, to study primate species beyond the great apes, the Japanese macaque was an appropriate candidate because it falls in Catarrhini, together with the apes [33]. Second, thanks to the extensive efforts of the troop managers to maintain detailed genealogical records, we could access comprehensive information on the birthdates and ages of all the subjects. This information is crucial for precise developmental studies. Third, this study required numerous facial photographs to examine temporal changes in facial development in detail. The free-ranging troop usually stayed in the feeding area with minimal hindrance during much of the day and were habituated to human observers, enabling us to collect an adequate number of images. Fourth, we selected subject animals within their native habitats rather than in captivity to ascertain the associations between infant faces and unrestricted behavior. As such, a free-ranging troop was an ideal subject for this study.

## Methods

### Study site and subjects

This study was conducted at Arashiyama Monkey Park Iwatayama, Kyoto, Japan. This private park has consistently fed one troop of free-ranging Japanese macaques (the Arashiyama troop) since 1954, presenting invaluable opportunities for visitors to observe wild monkeys [34]. Throughout this study, the Arashiyama troop consistently comprised approximately 130 monkeys. These macaques inhabit their native habitats, with unrestricted access to natural food resources and freedom in their behavior. The park has maintained genealogical records of these monkeys since the 1950s [34], availing accurate information on individual birthdays and ages.

The present study included 128 Japanese macaques, comprising 65 adult females and five adult males (≥ 5 years old), 25 juvenile females and 25 juvenile males (1−4 years old), and five infant females and three infant males (< 1 year old). All targets were monkeys of known age affiliated with this troop in 2021. Table 1 provides detailed information on the eight infant subjects: Chonpe’01’21, Cooper’90’01’21, Kusha’98’21, Cooper’94’02’21, Ai’02’21, Cooper’14’21, Cooper’94’01’21, and Cooper’89’02’21.

**Table 1:** Individual information on the eight Japanese macaque infants whose facial development and behavior were monitored.

Name^1^ (abbreviated name used in the text)	Sex	Maternal age	Maternal rank^2^	Siblings in the same troop
Chonpe’69’79’92’01’21 (Chonpe’01’21)	Female	20	Middle	2, 4 year old males
Cooper’65’71’90’01’21 (Cooper’90’01’21)	Female	20	Middle	3 year old male
Kusha’59’71’76’89’98’21 (Kusha’98’21)	Female	23	Middle	1, 2, 3 year old females
Cooper’65’81’94’02’21 (Cooper’94’02’21)	Male	19	Middle	(None)
Ai’61’72’83’96’02’21 (Ai’02’21)	Male	19	Low	3 year old male
Cooper’65’81’94’01’14’21 (Cooper’14’21)	Male	7	Middle	2 year old male
Cooper’65’81’94’01’21 (Cooper’94’01’21)	Male	20	Middle	1, 2, 8 year old males 7 year old female
Cooper’65’81’89’02’21 (Cooper’89’02’21)	Male	19	Middle	3 year old male, 8 year old female

^1^ The individual name has been determined by the mother’s name followed by the last two digits of the birth year of the offspring. For example, Chonpe’92’01’21 was born in 2021, her mother was born in 2001, and her grandmother in 1992. “Chonpe" is the name of the first generation.

^2^ The rank relationships were assessed based on their matrilineal lines.

### Morphometrics

Given that the research subjects were wild macaques, a non-contact method of measuring animal morphology was necessary to support the welfare of the subjects and minimize the ecological impact. Two non-contact methods have been employed to quantify wild primate faces: geometric morphometrics [24, 25] and allometry-based methods [9, 35, 36]. The former approach explores principal components elucidating the comprehensive facial morphology, with the distribution of the principal component scores subsequently used to attribute meaning to each principal component. The latter method employs allometric values of some facial parts to establish an infantile face scale, followed by an assessment of its scores. In this study, we used the latter approach so that we could objectively investigate the third question. In other words, we mitigated the risk of making arbitrary judgments potentially caused by initially checking the distribution of the principal component scores gained from the geometric morphometrics method and age before investigating the third objective.

The analysis focused on six facial features: face width relative to head length (FWHL), forehead length relative to face length (FoLFaL), eye width relative to face width (EWFW), nose length relative to head length (NLHL), nose width relative to face width (NWFW), and mouth width relative to face width (MWFW). These facial features have been regarded as typical infantile features in humans [9] and were discussed in a cross-species study [24]. Therefore, our methodology allowed for comparisons between the results from Japanese macaques and previous knowledge from other primates.

We collected facial images for 141 days, from December 2020 to August 2022. Notably, there was a brief intermission in data collection from April 25 to May 21, 2021, owing to the temporary closure of the park caused by the COVID-19 outbreak. To capture the photographic data, we used a digital camera (Canon EOS M6 with Canon EF–S55–250 mm F4–5.6 IS Stem lens) or a video camera (Panasonic HC-WZ590M-W) positioned at a distance exceeding 2 m from the subjects. Considering the focus on infant development, the frequency of capturing facial photographs differed according to the participant’s age. Adults were photographed once throughout the study period. Juveniles underwent photography approximately every three months from April 2021 to August 2022, with a maximum of four images per subject. For infants, images were captured weekly from birth to 24 weeks, followed by a transition to monthly intervals until the age of 1 year, yielding a maximum of 31 photographs per infant. In total, we captured 470 facial photographs: 70 from adults, 184 from juveniles, and 216 from infants.

**Fig 1:**
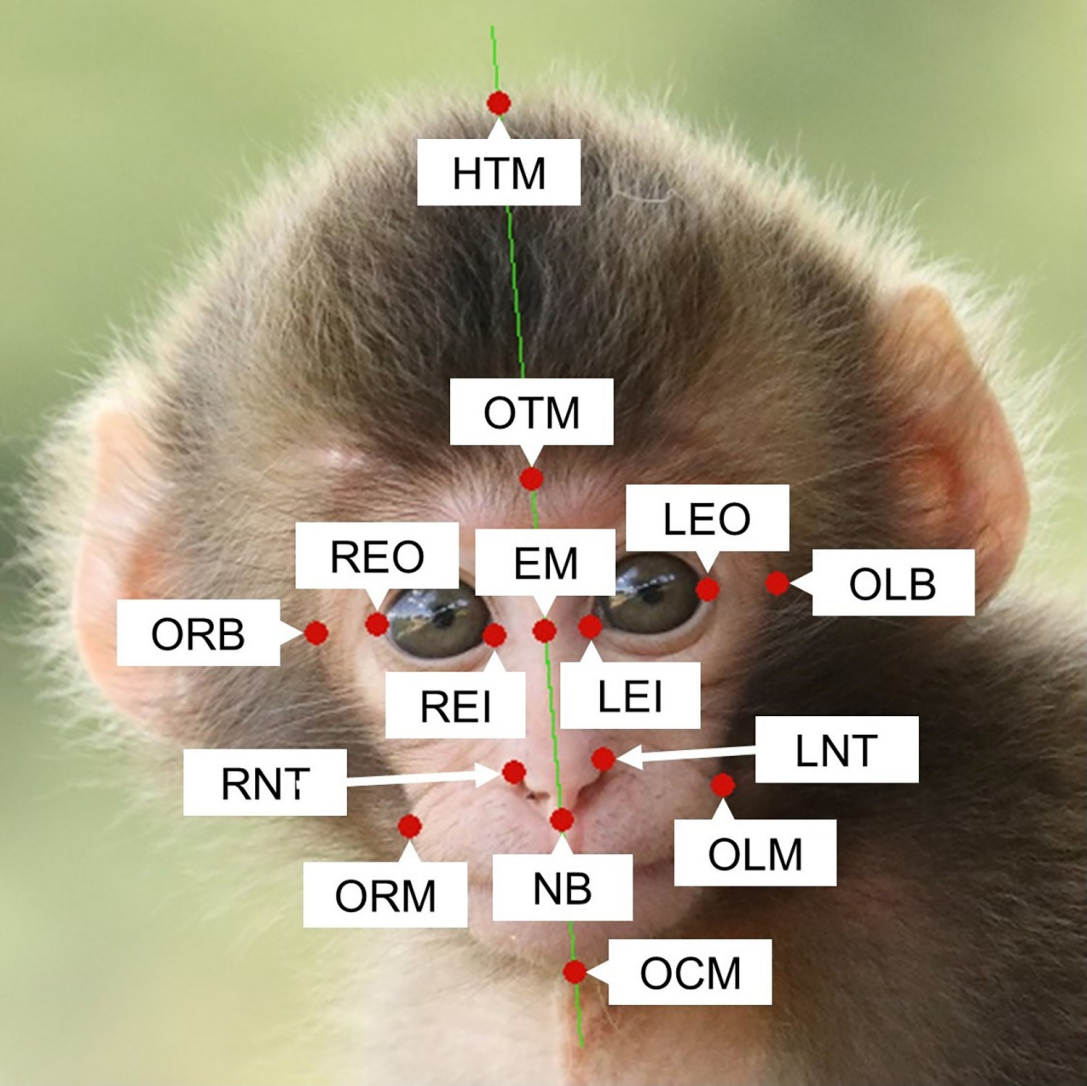
Example of landmark plots on the frontal face view of Japanese macaque infants.

GIMP (version 2.10.22: https://www.gimp.org/) was used for photographic analysis. All photos were cropped into a square format with dimensions of 500 × 500 pixels, which included the entire faces of the subjects. Subsequently, the first author manually plotted 15 landmarks on each photo (Fig 1 and Table 2). Following this process, the lengths of nine distinct facial parts (outlined in Table 3) were quantified in terms of pixels using the measure tool in GIMP. Finally, six target indices (FWHL, FoLFaL, EWFW, NLHL, NWFW, and MWFW; Table 4) were calculated.

**Table 2:** Definitions of Japanese macaque infant facial landmarks evaluated in this study.

Landmark	Abbreviation	Definition
HeadTop_Mid	HTM	Centre of top of the head
OutlineTop_Mid	OTM	Top center of the outline of the face
OutlineRight_Brow	ORB	Outline at right point horizontally aligned to eye outers
OutlineLeft_Brow	OLB	Outline at left point horizontally aligned to eye outers
OutlineChin_Mid	OCM	Point on the outline of the center of the chin
Eyes_Midpoint	EM	Point halfway between two eye inners on the midline of the face
RightEye_Inner	REI	Inner corner of the right eye
RightEye_Outer	REO	Outer corner of the right eye
LeftEye_Inner	LEI	Inner corner of the left eye
LeftEye_Outer	LEO	Outer corner of the left eye
OutlineRight_Mouth	ORM	Right corner of the mouth
OutlineLeft_Mouth	OLM	Left corner of the mouth
RightNostril_Top	RNT	Center of the top edge of the right nostril
LeftNostril_Top	LNT	Center of the top edge of the left nostril
Nostrils_Bottom	NB	Point halfway between the bottoms of two nostrils

The definitions were taken from the “primate_face” model of DeepLabCut Model Zoo (http://www.mackenziemathislab.org/dlc-modelzoo), and only some of the expressions were changed.

“Outline” means the edge of the hairless area.

**Table 3:** Facial parts and inter-rater reliability of the measurements.

Facial part	Abbreviation	Definition^1^	ICC (3,1)	95% CI	Assessment^2^
Head length	HL	HTM–OCM	0.89	0.85–0.93	Good–Excellent
Forehead length	FoL	HTM–OTM	0.98	0.98–0.99	Excellent
Face length	FaL	OTM–OCM	0.95	0.93–0.97	Excellent
Face width	FW	ORM–OLM	0.81	0.74–0.87	Moderate–Good
Right-eye width	REW	REI–REO	0.91	0.88–0.94	Good–Excellent
Left-eye width	LEW	LEI–LEO	0.90	0.86–0.93	Good–Excellent
Nose length	NL	EM–NB	0.97	0.96–0.98	Excellent
Nose width	NW	RNT–LNT	0.73	0.64–0.81	Moderate–Good
Mouth width	MW	ORM–OLM	0.83	0.76–0.88	Good

^1^ Distance between two facial landmarks

^2^ The reliability was assessed based on the guideline proposed by Koo and Li (2016): “Excellent” for 95% CI > 0.90, “Good” for 0.75 < 95% CI < 0.90, and “Moderate” for 0.50 < 95% CI < 0.75.

**Table 4:** Summary of the six facial measurement indices calculated for the analysis of facial development in Japanese macaque infants.

Index	Abbreviation	Formula
Relative size of face width	FWHL	FW/HL
Relative size of forehead length	FoLFaL	FoL/Fal
Relative size of eye width	EWFW	((REW + LEW)/2)/FW
Relative size of nose length	NLHL	NL/HL
Relative size of nose width	NWFW	NW/FW
Relative size of mouth width	MWFW	MW/FW

To ascertain the reliability of the measurements performed by the first author, an additional rater, who was blinded to the study aims and had limited familiarity with Japanese macaques, placed landmarks on a subset of 118 images (25.1% of the total), including 18 adults, 46 juveniles, and 54 infants. Using these landmarks, we computed nine facial parts (Table 3) and evaluated their consistency with the measurements obtained by the first author using the intraclass correlation coefficient (ICC). This study focused on the reliability of the relative relationships among the measured values. To this end, a two-way mixed effects, single rater ICC (ICC (3,1)) was calculated using R studio version 4.2.2 (R Core Team, 2022) and the "psych" package (ver. 2.3.9). The reliability evaluation was based on 95% confidence intervals [37], and the outcomes revealed almost satisfactory matches (Table 3 and S1 Fig). Consequently, we used the measurements from the first author for subsequent analyses.

### Behavioral observations

We conducted two types of behavioral observations to investigate caretaking behaviors directed toward the target infants and examined the development of their motor skills. For recording caretaking behaviors, two female (Chonpe ’01’21, Kusha ’98’21) and two male infants (Cooper ’94’02’21, and Cooper ’94’01’21) were selected from the eight included in this study. These four individuals were born after the park-closure period and had minimal missing facial photograph data. We used focal animal sampling [38] between August and December 2021, with each session lasting 10–30 min, when the target infants were 12–23 weeks of age. This age corresponds to the period when infants are often separated from their mothers and start to actively engage with other troop members. The indices focused on were the duration of affiliative physical contact with the mother or other troop member, including cuddling, grooming, carrying, and learning. The average observation duration per infant was 495.0 minutes (*SD* = 16.6).

Furthermore, we studied the developmental stages of all eight infants to record their motor skills and exploratory behaviors. The target behaviors were the five behavioral milestones linked to mobility and exploration in primates (Table 5). We recorded the presence or absence of these milestones in each subject while collecting facial photographs. Behavioral monitoring was initiated immediately after birth and persisted until each milestone was observed at least once in every infant.

**Table 5:** Description of the five behavioral milestones observed during Japanese macaque infant development.

Behavioral milestones	Definition
Walk	Infants walk independently.
> 2 m away from the mother	Infants move > 2 m away from their mothers.
> 5 m away from the mother	Infants move > 5 m away from their mothers.
> 10 m away from the mother	Infants move > 10 m away from their mothers.
Social play	Infants play with other monkeys, including chasing and wrestling.

### Data analysis

R Studio (version 4.2.2) was used for all statistical analyses. To prepare the data for analysis, individual Z-scores were computed for each facial feature. This involved subtracting the overall means from the individual measurements and dividing them by the standard deviations. The resulting Z-scores were used as the basis for subsequent statistical assessments.

#### (1) Identifying infantile facial features and quantifying infantile faces

First, to examine infantile facial features in Japanese macaques, linear mixed models using the "glmmTMB" package (ver. 1.1.3) were used to compare the Z-scores for each facial index across three age categories: infants, juveniles, and adults. The response variable was each individual’s facial index, the explanatory variables included age category and sex, and the random effect was individual ID. In order to verify the multicollinearity, we checked whether variance inflation factors (VIF) exceeded 3 [39] using the "performance" package (ver. 0.10.5) [40]. Next, using the Akaike information criterion (AIC), we compared the model fit between the model with all explanatory variables (full model) and that without them (null model). We selected the model with the smallest AIC value as the final model [41]. In cases where age exhibited a significant association with the explanatory variable, we conducted multiple comparisons by the Tukey method using the "multcomp" package (ver. 1.4.25).

Next, we defined infantile face scores (IFS) for Japanese macaques. This variable was referenced from Glocker et al. [9] and indicates the mean value of the Z-scores of all infantile facial features ascertained in this study. Prior to IFS calculation, sign reversal was applied to facial features with smaller Z-scores for infants. This methodology provided us with a valid means of quantifying infantile faces of Japanese macaques.

#### (2) Association between infantile faces and caretaking behaviors

To examine the link between infantile faces and caretaking behaviors toward infants, we performed generalized linear mixed models using the "glmmTMB" package. The explanatory variable was the total duration of affiliative contact with the mother or non-mother (in integer seconds) recorded on each observation day. The explanatory variables were the IFS, age (in days) on the day of observation, and sex. The IFS were the measured values of the photos taken on the day closest to the date of behavioral recording. Moreover, we introduced each day’s observation duration (in min) as an offset term. Because the response variables included numerous zeros (16/80 for mothers and 41/80 for non-mothers), we fitted four potential distributions as error structures: Poisson distribution, negative binomial distribution, zero-inflated Poisson distribution, and zero-inflated negative binomial distribution. Null models were also constructed for Poisson and negative binomial distributions. The model with the smallest AIC value was selected as the final model. To check multicollinearity, VIF was computed with the "performance" package (ver. 0.10.5). In cases where the highest 95% confidence interval of the VIF exceeded 3, we excluded the variable with the highest VIF, other than IFS, from the models.

#### (3) Developmental process of infantile faces during infancy

Using the IFS, we explored the development of infantile faces within the first 24 weeks of age. To effectively examine the potential non-linear development of infantile faces, we formed three types of regression models and compared their fitting. The first was a linear model, the second was a linear model with the squared term for age as an additional explanatory variable, and the third was a generalized additive model (GAM). The smoothing parameter for the GAM was the generalized cross-validation method. In these models, IFS was the response variable and age (in days) was the explanatory variable. We used the "lm" function for the linear models and the "gam" function from the "mgcv" package (ver. 1.9.0) for GAM. Among the three candidate models and the null model, the model with the smallest AIC value was chosen as the final model for each individual. When multiple models demonstrated equal AIC values, the simpler model was selected as the final model.

#### (4) Association between infantile facial development and mobility development

Finally, to explore the associations between IFS development and infant mobility, we created figures showing the dates of the initial observation of five behavioral milestones and the date of the estimated IFS peak within the first 24 weeks of life [42]. Note that the behavioral milestone data only indicate the date the milestones were first observed, and it is possible that the infants may have exhibited the behavior prior to our first records. Additionally, because of the study interruption between April and May 2021, continuous recordings were stopped for four infants (Ai’02’21, Cooper’90’01’21, Cooper’89’02’21, and Cooper’14’21; missing observations at 11–37, 0–18, 0–17, and 0–14 days of age, respectively). Therefore, we excluded parts of the data from these four individuals from the analysis. Specifically, we evaluated whether the age at which the corresponding behavioral milestone was initially observed in other infants overlapped with or was less than the unobservable age in each of the four infants. If such overlap or earlier records in other infants were found, the data on that milestone for that infant were excluded from the analysis. This led to the exclusion of four behavioral milestones (observed at least 2 m away, 5 m away, and 10 m away from the mother, and social play) for Ai’02’21 and two behavioral milestones (walking and being at least 2 m away from the mother) for Cooper’90’01’21, Cooper’89’02’21, and Cooper’14’21 from the analysis.

### Ethics statements

The study adhered to the Guidelines for Field Research established by the Ethics Committee of the Primate Research Institute, Kyoto University. The Arashiyama Monkey Park Iwatayama preapproved this study.

## Results

### Identifying infantile facial features and quantifying infantile faces

**Fig 2:**
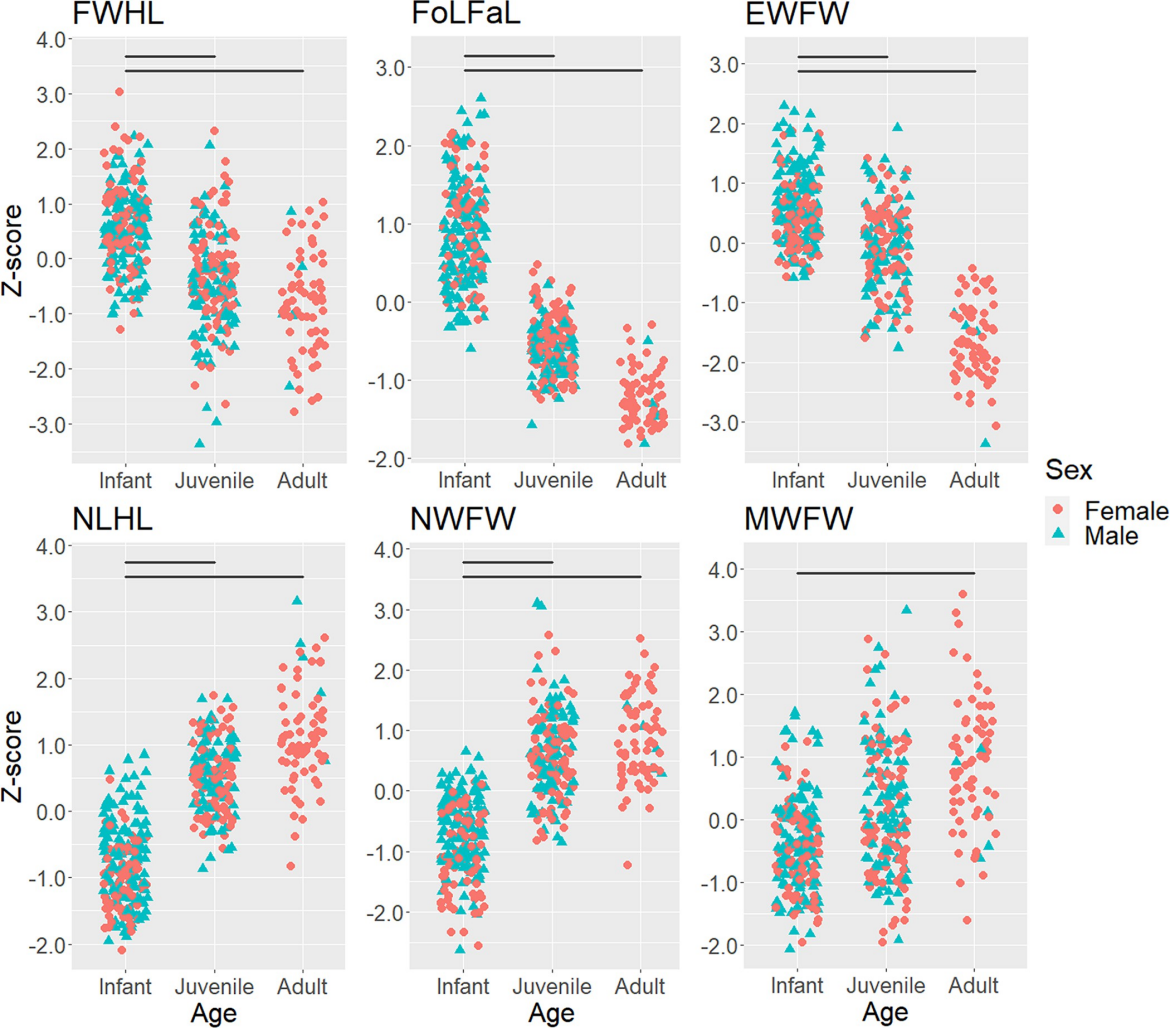
Comparisons of each facial feature between infant, juvenile, and adult Japanese macaques. Horizontal lines indicate significant differences between age classes. See text for significance levels.

For all facial features, the AIC of the full model examining infantile features was smaller than that of the null model (S1 Table), resulting in the selection of the full model for all features. The VIF of the explanatory variables remained below 3 in all six full models, showing that there was no multicollinearity among the explanatory variables. For all facial features, age categories showed significant associations with the response variables (Table 6). Multiple comparisons revealed that in FWHL, FoLFaL, and EWFW, infants exhibited significantly higher Z-scores than both juveniles (FWHL: *p* = 2.59e-05, FoLFaL: *p* < 2e-16, EWFW: *p* = 0.000136) and adults (FWHL: *p* = 4.43e-09, FoLFaL: *p* < 2e-16, EWFW: *p* < 2e-16). In contrast, the Z-scores of NLHL and NWFW were significantly lower in infants than in juveniles (NLHL: *p* < 2e-16, NWFW: *p* = 1.20e-14) and adults (NLHL: *p* < 2e-16, NWFW: *p* < 2e-16). In the context of the MWFW, infants exhibited significantly lower Z-scores than adults (*p* = 8.46e-06) and tended to have smaller Z-scores than juveniles (*p* = 0.0513). These findings indicate that infants showed significantly different values compared to adults and approximately distinct values compared to juveniles for all six indices (Fig 2). Consequently, we judged all six indicators as infantile facial features in Japanese macaques.

**Table 6:** GLMM results comparing the facial index between age categories in Japanese macaques.

(1) FWHL	Estimate	Std. Error	*z* value	Pr (> |*z*|)
(Intercept)	0.7194	0.2299	3.130	0.00175 **
AgeJuvenile	-1.0156	0.2287	-4.441	8.97e-06 ***
AgeAdult	-1.4960	0.2479	-6.034	1.60e-09 ***
SexMale	-0.2212	0.1593	-1.388	0.16503
(2) FoLFaL	Estimate	Std. Error	*z* value	Pr (> |*z*|)
(Intercept)	0.97358	0.05710	17.049	< 2e-16 ***
AgeJuvenile	-1.49627	0.05972	-25.053	< 2e-16 ***
AgeAdult	-2.19809	0.08245	-26.661	< 2e-16 ***
SexMale	-0.12409	0.05803	-2.138	0.0325 *
(3) EWFW	Estimate	Std. Error	*z* value	Pr (> |*z*|)
(Intercept)	0.592916	0.162665	3.645	0.000267 ***
AgeJuvenile	-0.657223	0.161729	-4.064	4.83e-05 ***
AgeAdult	-2.265101	0.177775	-12.741	< 2e-16 ***
SexMale	-0.001261	0.116403	-0.011	0.991354
(4) NLHL	Estimate	Std. Error	*z* value	Pr (> |*z*|)
(Intercept)	-1.0110	0.1635	-6.182	6.32e-10 ***
AgeJuvenile	1.4221	0.1625	8.749	< 2e-16 ***
AgeAdult	2.1495	0.1770	12.144	< 2e-16 ***
SexMale	0.2668	0.1147	2.326	0.02 *
(5) NWFW	Estimate	Std. Error	*z* value	Pr (> |*z*|)
(Intercept)	-0.8849	0.1833	-4.829	1.37e-06 ***
AgeJuvenile	1.4263	0.1823	7.823	5.14e-15 ***
AgeAdult	1.7261	0.1972	8.754	< 2e-16 ***
SexMale	0.1394	0.1263	1.104	0.27
(6) MWFW	Estimate	Std. Error	*z* value	Pr (> |*z*|)
(Intercept)	-0.4520	0.2632	-1.717	0.0859
AgeJuvenile	0.6065	0.2619	2.316	0.0206 *
AgeAdult	1.3166	0.2820	4.669	3.03e-06 ***
SexMale	0.0420	0.1795	0.234	0.8150

* < .05

** < .01

*** < .001

**Fig 3:**
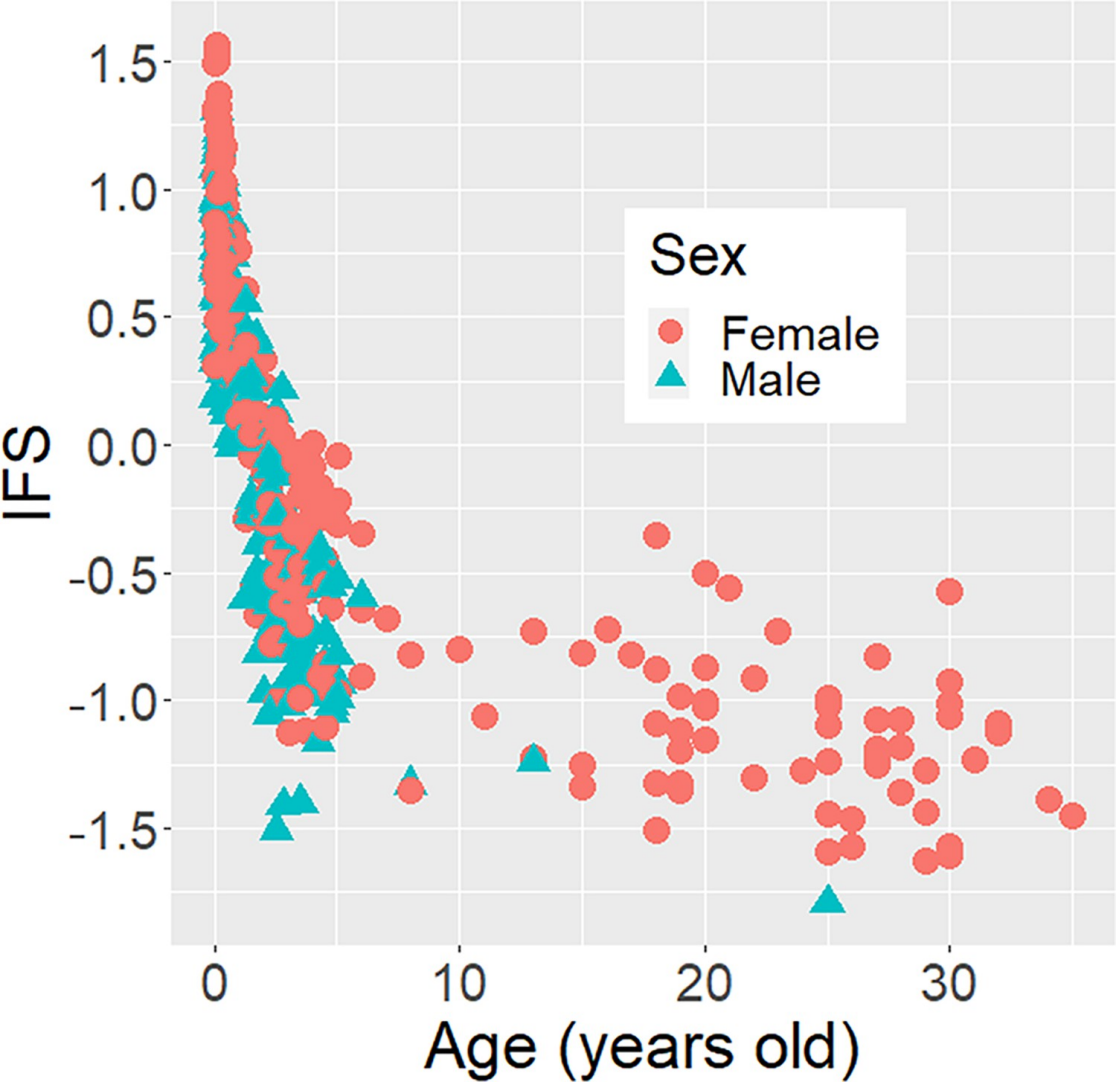
Developmental changes of IFS in Japanese macaques of all ages. The ages of adults were included as whole numbers (years, integers), while the ages of infants and juveniles were included as their age in days divided by 365.

These findings defined IFS as the mean value of FWHL, FoLFaL, EWFW, NLHL, NWFW, and MWFW. In calculating the IFS, we reversed the signs of NLHL, NWFW, and MWFW values, because the smaller values indicate more infantile traits for those three, contrary to the other three. When plotting IFS with age, it apparently decreased from early life to adulthood (Fig 3), indicating that IFS is a robust quantitative measure for capturing the infantile faces of Japanese macaques.

### Associations between infantile faces and caretaking behaviors

Among the models of the total duration of affiliative contact with the mother, the zero-inflated negative binomial model showed the smallest AIC value (S1 Table). However, the highest 95% confidence interval of the VIF for age was 5.03, indicating multicollinearity. Therefore, we formulated models with each error distribution, excluding age. Among them, the AIC was also the smallest in the model with the zero-inflated negative binomial distribution (S1 Table), and the VIF for this model was below 3. The results indicated the absence of a significant association between the IFS and the duration of affiliative contact with the mother (Table 7).

**Table 7:** GLMM results examining the association between IFS and affiliative contact duration with caregivers of Japanese macaque infants.

(1) Affiliative contact duration with the mother				
	Estimate	Std. Error	*z* value	Pr (> |*z*|)
(intercept)	3.28446	0.40567	8.096	5.66e-16 ***
IFS	0.39495	0.48319	0.817	0.414
SexMale	0.05267	0.24366	0.216	0.829
(2) Affiliative contact duration with nonmothers				
	Estimate	Std. Error	*z* value	Pr (> |*z*|)
(intercept)	1.99018	0.42430	4.69	2.73e-06 ***
IFS	0.01227	0.60547	0.02	0.984

In the model examining the total duration of affiliative contact with non-mothers, the zero-inflated negative binomial model showed the smallest AIC value (S1 Table). However, the highest 95% confidence interval of VIF for IFS and sex were 5.94 and 5.74, respectively. Thus, sex variables were excluded from each model. Subsequently, the negative binomial model was again selected by AIC (S1 Table), but the highest 95% confidence interval of VIF for IFS and age was very high (1.83 × 10^5^). Therefore, age was excluded from our model. Finally, among the models with only the IFS as an explanatory variable, the model with a zero-inflated negative binomial distribution displayed the smallest AIC value (S1 Table). The results demonstrated that the IFS was not significantly associated with the duration of affiliative contact with non-mothers (Table 7).

### Developmental process of infantile faces during infancy

**Fig 4:**
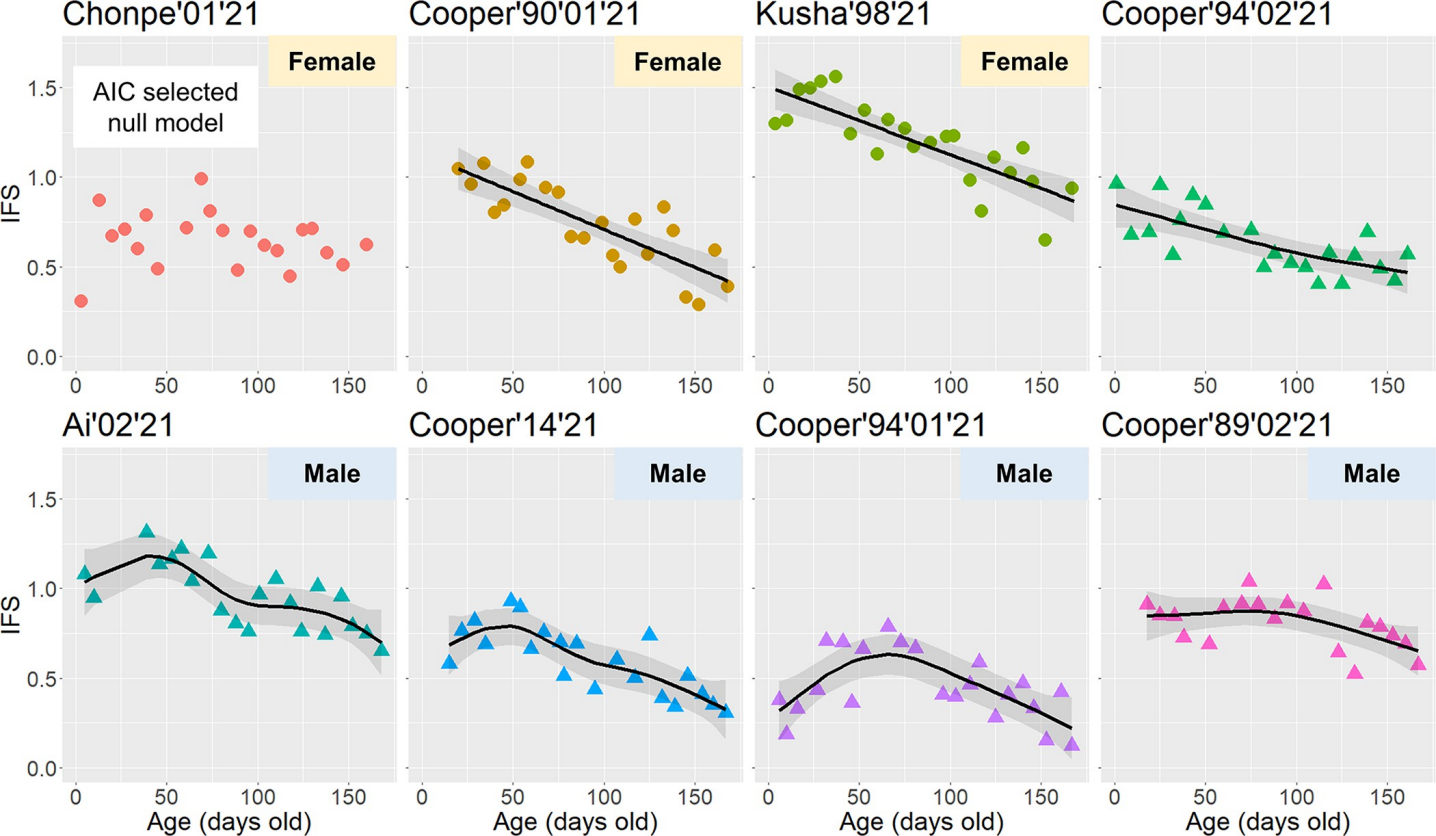
IFS development of each infant within the first 24 weeks of life. Black lines and gray ribbons represent the estimated values and 95% confidential intervals. The circles and triangles indicate females and males, respectively.

**Fig 5:**
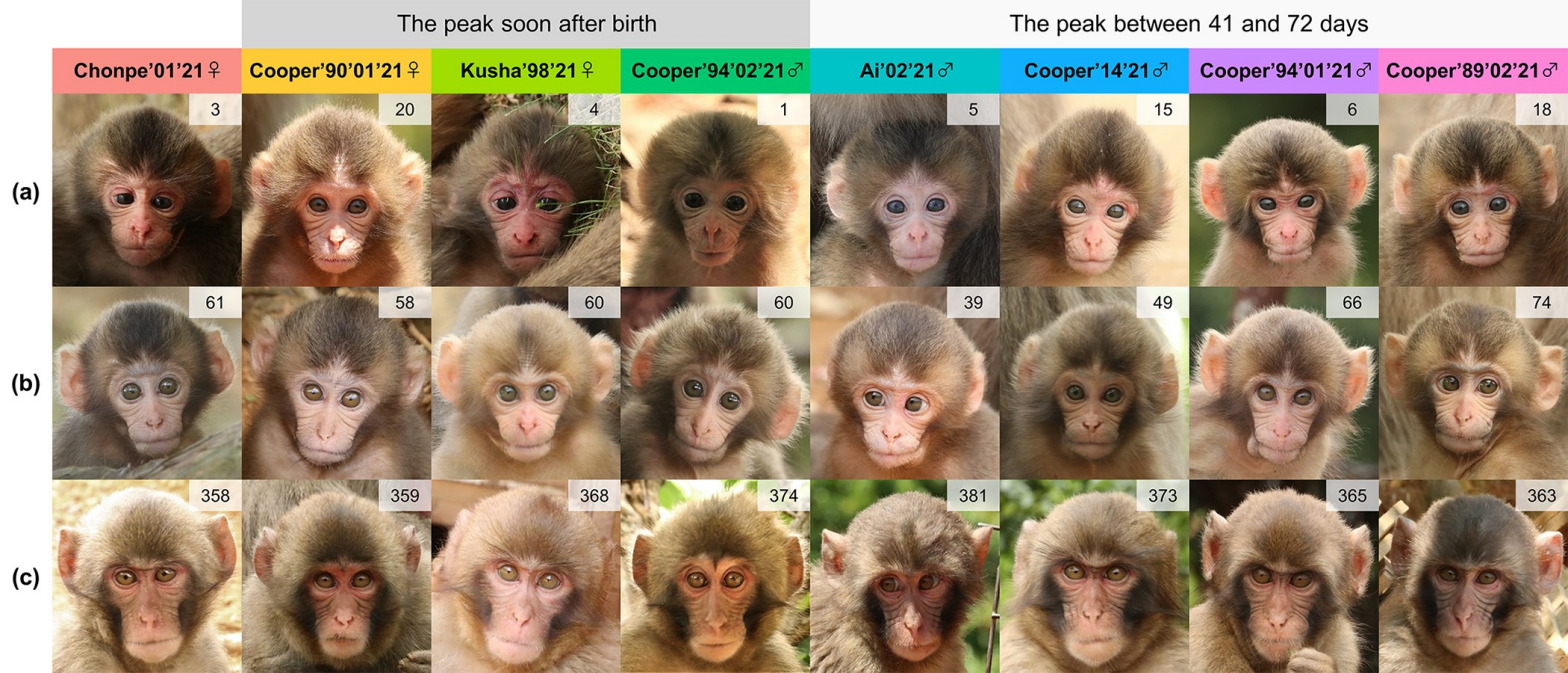
Facial appearance of each infant at three different stages: (a) newborn, (b) between approximately 41 to 72 days of age, when IFS peaked for some infants, and (c) around 1 year after birth. In (a), the youngest facial photographs of each infant were shown.

After formulating four candidate models, the AIC selected the linear model for Cooper’90’01’21 and Kusha’98’21, GAM for Cooper’94’02’21, Ai’02’21, Cooper’14’21, Cooper’94’01’21, and Cooper’89’02’21, and the null model for Chonpe’01’21 (S1 Table). Based on these outcomes, we found two distinct patterns of IFS development (Fig 4). Three infants (Cooper’90’01’21, Kusha’98’21, and Cooper’94’02’21) showed consistent decreases in IFS until 24 weeks of age. Conversely, although the 95% confidence intervals were wide, the IFS of four individuals (Ai’02’21, Cooper’14’21, Cooper’94’01’21, and Cooper’89’02’21) increased immediately after birth and then decreased. The peak IFS estimates for these four infants were 41.22, 45.71, 66.17, and 72.18 days, respectively. Notably, the three infants with consistently decreasing IFS included two females and one male, whereas all four infants with a hump-shaped IFS development were male (Fig 4). Based on these results, Fig 5 shows the facial photographs of each infant at three ages: newborns, between approximately 41 to 72 days of age, and about 1 year after birth.

### Association between infantile facial development and mobility development

**Fig 6:**
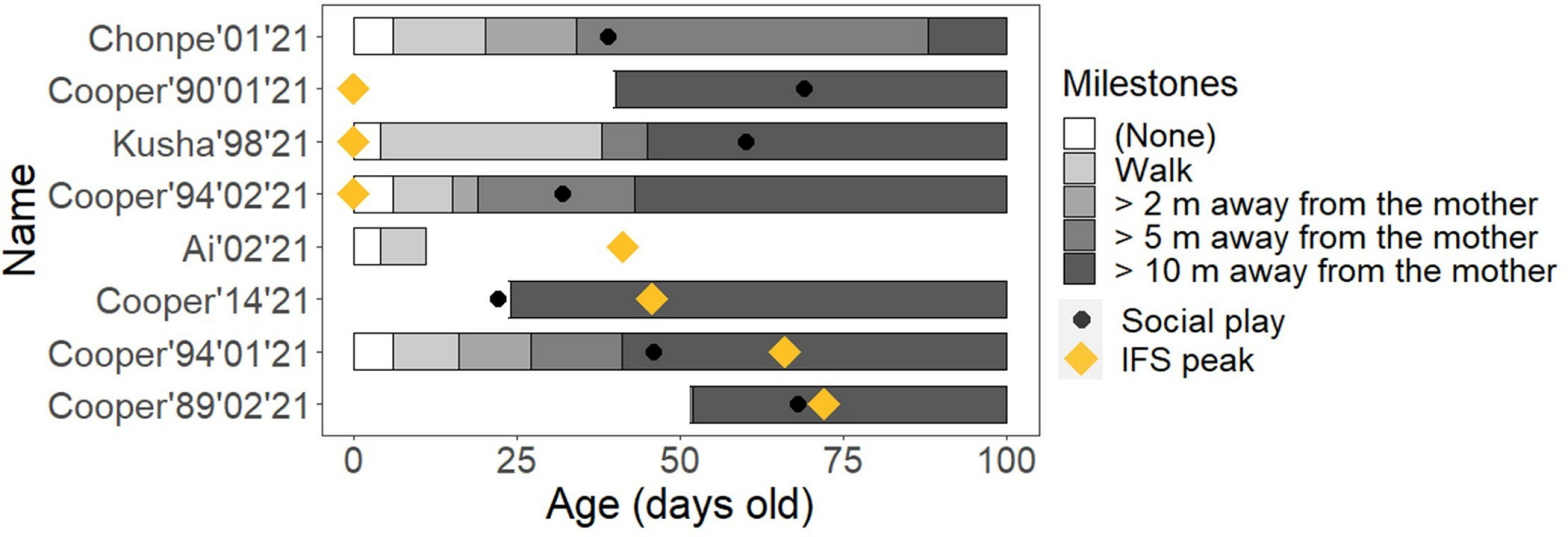
The age with estimated IFS peaks and the first-observed age in days of the five behavioral milestones in the eight Japanese macaque infants observed. Certain information is missing due to the temporary suspension of observations.

Fig 6 depicts the association between the age of the estimated IFS peak for each infant and the behavioral milestones. We found no consistent trend between the IFS and infant mobility or exploratory behavior.

## Discussion

### Infantile facial features and their association with caretaking behaviors

The first aim of this study was to ascertain the infantile facial features of Japanese macaques. The findings revealed significant differences between the infants and adults across all six indices. Moreover, a consistent trend was observed in Japanese macaques and humans, characterized by larger face width, forehead length, and eye width, along with a smaller nose and mouth. Therefore, Japanese macaques have these six indices as infantile facial features, similar to humans. Some features, such as face width, forehead length, and eye width, have also been reported as shared infantile features among great apes [24], suggesting the likelihood that these infantile facial features are shared at least among Catarrhini.

We also found differences between the infantile facial features of Japanese macaques compared to great apes. Kawaguchi et al. [24] deduced that the sizes of the nose and mouth were not shared infantile features among great apes. In contrast, we found that nose and mouth sizes were associated with infantile features in Japanese macaques. The differences between these studies could be attributed to two factors. First, there may be species differences in infantile facial features [24, 25, 43]. For example, infant chimpanzees have rounded supraorbital torus as infantile features that have not yet been reported in humans [25]. Differences in the nose and mouth size between great apes and Japanese macaques may also reflect interspecific differences. If so, the interspecies variation in infantile features may surpass what Lorenz [8] initially assumed. Second, the methodological differences between our study and Kawaguchi et al. [24] may have contributed to this variation. This study directly measured candidate facial features, whereas Kawaguchi et al. [24] used geometric morphometrics. This methodological difference potentially accounts for the heightened sensitivity to the nose and mouth size in our study. Future studies should consider these methodological differences to extend the research to a broader range of species to reveal the interspecific similarities and variations in infantile facial features.

The second objective was to examine whether infantile faces in Japanese macaques exhibit a notable link with caretaking behaviors toward infants. We did not find significant associations between infantile faces and affiliative contact with mothers or non-mothers. This finding is consistent with that of Koda et al. [29], who reported no preference for infant faces in this species. Additionally, when exploring the responses to infantile faces in some great apes, chimpanzees displayed a significant but very weak preference and bonobos exhibited none [25, 28]. In contrast, infantile faces in humans can elicit strong responses and may facilitate caretaking motivation and affectionate care [9–12, 18, 19]. Contrary to Lorenz [8], these results suggest significant species differences in the link between infantile facial shape and caretaking behaviors toward infants, with a much stronger link in humans.

The underlying factors contributing to the heightened reactivity in humans to infantile faces remain unclear, but several possibilities exist [44, 45]. For example, Glocker et al. [18] emphasized the importance of a higher frequency of non-maternal caretaking behaviors. In cooperative breeding species like humans [6], non-mothers’ preference for shared infantile facial features within the species may provide adaptive advantages to them. Although Barbary macaques [26] perform triadic male-infant interactions, called “bridging” [46], studies of non-human primates, including ours, have not yet included species that engage in cooperative breeding or exhibit frequent non-maternal caretaking. Therefore, to test this hypothesis, it is imperative to examine how other cooperative breeding primates, such as Callithrichidae [47–49], respond to infantile facial features.

As another possibility, interactions with other infantile physical features, such as the body or fur color specific to infants (known as “infantile coloration”), may also hold significance. Chimpanzees exhibit a stronger response to infantile facial skin color than to facial shape [25], whereas humans lacking distinct infant coloration [45] display a stronger response to facial shape [9–12]. This suggests a trade-off between increased reactivity to infantile coloration and stronger reactions to infantile facial morphology. Japanese macaque infants exhibit pinkish skin and dark hair colors, unlike adults. Therefore, body color, rather than facial shape, may draw the attention of surrounding individuals in this species. However, this trade-off explanation is inconsistent with the finding that bonobos, which lack infantile coloration, did not prefer infant images to those of adults [28]. Further comparative studies are warranted to determine whether and how infantile coloration affects reactivity to infantile facial shape among primates.

Note that the response of Japanese macaques to infantile faces remains a topic without conclusive evidence. To the best of our knowledge, Koda et al. [29] and the present study are the only empirical investigations exploring the connection between infant faces and the responses of Japanese macaques. However, both studies were conducted with limited sample sizes, consisting of only two nulliparous females in Koda et al. [29] and four infants in the current study. Additionally, the behavioral observations employed in our study were simplified; we could not target potentially associated behaviors other than affiliative contact and direct care, such as the interest in infants [50–52] or careful behavioral responses [20, 53, 54]. Furthermore, we could not control for other potential factors affecting caretaking behavior in Japanese macaques, such as maternal age, parity [55, 56], and social relationships between non-maternal caregivers and mothers [57]. Therefore, future research should include more precise observational approaches and statistical designs to further examine the responses to infantile faces among primates.

### Development of infantile faces and associations with mobility

The third objective was to investigate the development of infantile faces in Japanese macaques during the early postnatal period. Although studies on non-human animals have explored the developmental process of infantile coloration during infancy [58, 59], this study represents the first empirical example of the development of infantile faces and their developmental patterns in non-human animals. Our findings revealed that infantile faces did not necessarily reach their peak proportions immediately after birth but instead peaked between 41 and 72 days of age in some subjects. This hump-shaped developmental trajectory of infantile faces parallels findings in humans [16, 21, 22, 23]. However, since we could only access the averaged results from previous studies in humans, it remains uncertain whether human infants also have particular patterns in infantile facial development or whether the hump-shaped developmental trajectory is more commonly observed in humans. The hump-shaped development of infantile faces may also be found in other animal species such as chimpanzees, dogs, cats, and rabbits, because human responses to images of these animals also exhibit a hump-shaped form with increasing age of the stimulus [23]. Research on primates and other animals, including humans, is essential for understanding the variations in the development of infantile faces within and across species.

Our data implicated sex as a factor in the two developmental patterns observed in infantile faces. Four out of the five males included in the study exhibited a hump-shaped development, whereas all three females did not. Sex differences in adult facial appearance are associated with androgens and other hormones in humans [60, 61]. Similar mechanisms might have underlain the present findings. For instance, testosterone levels, a kind of androgen, may be higher in male than in female macaque neonates [62], which could cause sex-based differences in the appearance of infantile faces.

As an important study on sex differences, Hamada et al. [63] demonstrated that facial measurement data from over 3,000 Japanese macaques, including 377 monkeys less than 6 months old, revealed no significant early postnatal sex-based differences in head or face length or width. Similarly, in other macaques, sex-based differences in craniofacial appearance are minimal in the early postnatal period and become more pronounced with maturity [64, 65]. In addition, although brain volume in rhesus monkey infants was consistently larger in males than in females, the difference was small [66, 67]. Thus, it is noteworthy that the sex-based trends found in this study represent very minor differences compared to those of adults. Moreover, we cannot exclude the possibility that the sex-based differences observed in this study were due to chance because the study cohort only comprised eight infants. The two patterns observed in infantile facial development may simply be the result of other factors, as discussed below. A more robust study with a larger cohort of infants is warranted.

Another possible factor of the two developmental patterns is the health status of the infants. In humans, infant health conditions may manifest as changes in facial appearance [68]. If a similar phenomenon was observed in Japanese macaques, the development of infantile faces could be partly affected by early postnatal health. Given that our study did not record detailed behavioral data of infants in the early postnatal period, this aspect warrants further exploration in future studies.

Inadequate sampling frequencies might have driven the current results. Although GAM analyses did not detect hump-shaped IFS development, a closer look at the plots in Fig 4 suggests that Chonpe’01’21 and Cooper’90’01’21 showed peaks at around 60 days of age, and Kusha’98’21 at around 40 days of age. The sampling interval of once a week might not have adequately captured developmental changes in each infant. By increasing the sampling frequency, the hump-shaped development of infantile faces may be commonly detected in Japanese macaque infants.

The fourth purpose of our study was to investigate whether infantile faces in Japanese macaques reach their peak at the onset of exploratory behavior. The hump-shaped development of infant faces might be linked to the function of capturing and retaining caregivers’ attention [16]. If infantile faces in non-human animal species also promote attention or caretaking behaviors, it would be valuable to examine this hypothesis in these species too. The current study did not demonstrate clear associations between peak IFS and the development of exploratory behaviors in Japanese macaques, although we found humped-shaped development of infantile faces in male individuals. This result was reasonable, given that infantile faces were not significantly associated with caretaking behaviors. Even in humans, the association between facial development and infant behaviors remains almost untested. Therefore, further research is warranted to examine the relationship between the development of infantile faces and infant behavior.

### Future directions

This study demonstrates that infantile facial features and the developmental patterns of infantile faces in Japanese macaques may exhibit trends similar to those of humans or non-human great apes, while also revealing partial species differences. Furthermore, we did not find any significant associations between infantile faces and behavioral indicators, as is the case in humans. Consistent with previous studies, these results suggest that although many morphological aspects of facial infantile-ness may be common across primates [8, 24], there are interesting interspecific variations in responses to these features, with particularly strong responsiveness in humans [9, 26, 28, 29]. Future research should target a broader range of lineages to further elucidate both the commonalities and variations in infant faces and the responses toward them.

Finally, as a prospective avenue for primate research, we emphasized the significance of physical features beyond frontal faces, such as profile, whole body, and body color. Although previous studies focused on frontal faces, Lorenz [8] originally suggested the role of other physical features in eliciting care among animals. Indeed, early studies in humans have indicated connections between infantile profiles or full-body images and perceptions of cuteness by observers [10, 11, 69]. Japanese macaques also demonstrate a preference for full-body images of conspecific infants [70]. In addition, many primates exhibit specific infantile coloration [45, 71], which may stimulate adult interest and caretaking behaviors [17, 25, 71–74]. In real-world caretaking contexts, caregivers have abundant exposure to various infantile physical features that are not limited to faces alone. Therefore, future research should include physical features beyond frontal facial characteristics to enable more detailed comparative studies of infantile physical features and the responses to them.

## Supporting information

S1 Fig Plots of the measurements of nine Japanese macaque facial parts from 118 photographs measured by two raters to assess inter-rater reliability. (DOCX)

S1 Table AIC results for the model selection in this study. (DOCX)

S1 File Raw data files for all analyses in this study. (XLSX)
