# Correction: Examining infantile facial features and their influence on caretaking behaviors in free-ranging Japanese macaques (*Macaca fuscata*)

**DOI:** 10.1371/journal.pone.0320027

**Published:** 2025-03-05

**Authors:** Toshiki Minami, Takeshi Furuichi

The funding statement for this paper is incorrect. The correct Funding statement is as follows: This study was financially supported by JSPS KAKENHI Grant Number JP24KJ1355.
