# Supplementary material for: Examining infantile facial features and their influence on caretaking behaviors in free-ranging Japanese macaques (Macaca fuscata)
Source: PLoS One. 2024 Jun 20;19(6):e0302412. doi: 10.1371/journal.pone.0302412 (PMC11189181; doi:10.1371/journal.pone.0302412)
Supplement: S1 Fig — (DOCX) [file pone.0302412.s001.docx]

*PLOS ONE*

Research Article

**Examining infantile facial features and their influence on caretaking behaviors in free-ranging Japanese macaques (*Macaca fuscata*)**

Short title: Infantile facial features and their behavioral effects in Japanese macaques.

Toshiki Minami^1*^, Takeshi Furuichi^2^

^1^ Graduate School of Education, Kyoto University, Kyoto, Kyoto, Japan

^2^ Wildlife Research Center, Kyoto University, Inuyama, Aichi, Japan

^*^ Corresponding author

E-mail: minami.toshiki.373@gmail.com

ORCID: 0000-0001-5476-3896

S1 Fig. Plots of the measurements of nine Japanese macaque facial parts from 118 photographs measured by two raters to assess inter-rater reliability.


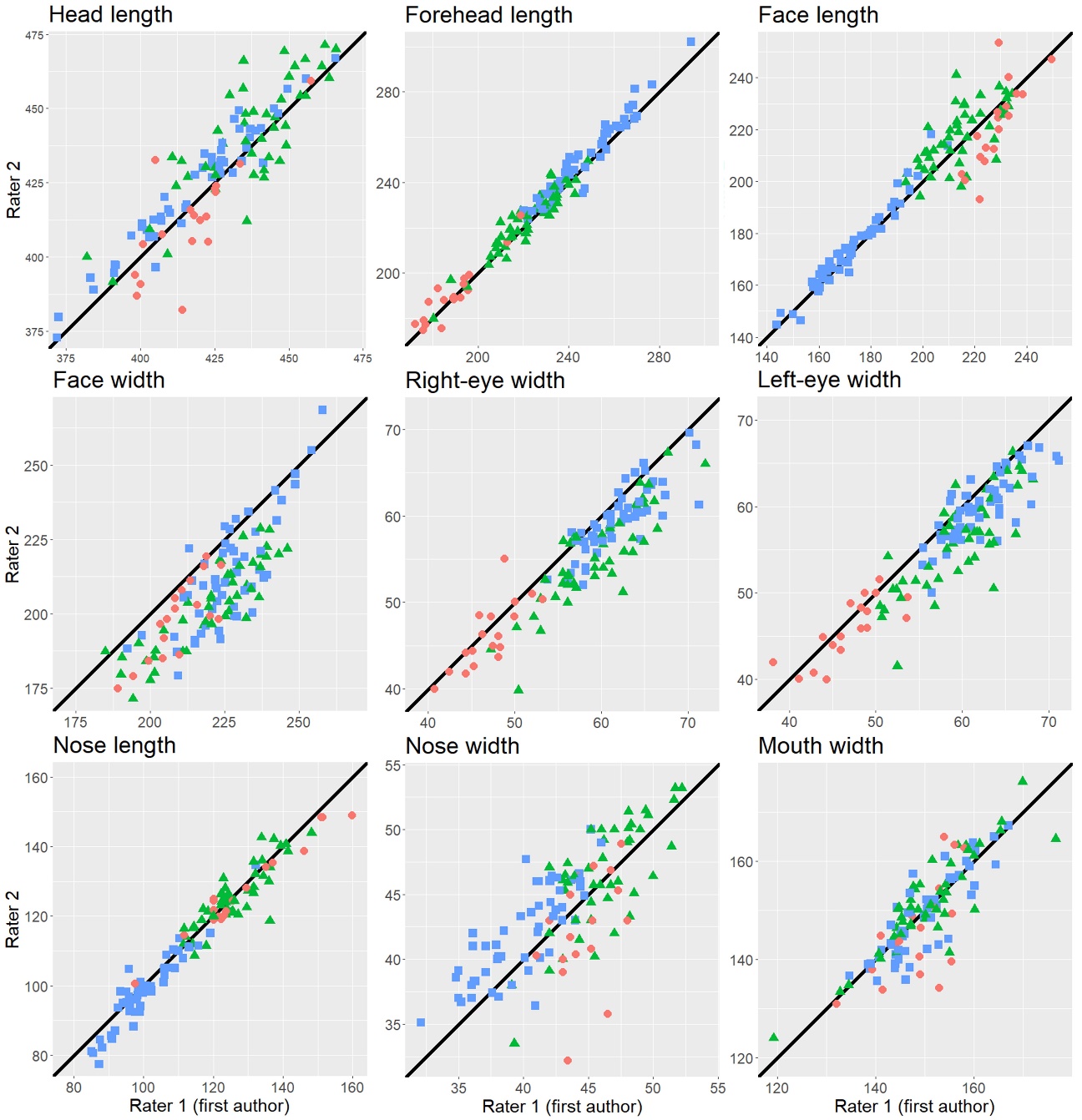


Note) The color-coded shapes, such as orange circles, green triangles, and blue squares, correspond to adult individuals, juveniles, and infants, respectively. The black lines depict instances where the ratings given by two raters precisely matched (y = x).
