# Supplementary material for: Examining infantile facial features and their influence on caretaking behaviors in free-ranging Japanese macaques (Macaca fuscata)
Source: PLoS One. 2024 Jun 20;19(6):e0302412. doi: 10.1371/journal.pone.0302412 (PMC11189181; doi:10.1371/journal.pone.0302412)
Supplement: S1 Table — (DOCX) [file pone.0302412.s002.docx]

*PLOS ONE*

Research Article

**Examining infantile facial features and their influence on caretaking behaviors in free-ranging Japanese macaques (*Macaca fuscata*)**

Short title: Infantile facial features and their behavioral effects in Japanese macaques.

Toshiki Minami^1*^, Takeshi Furuichi^2^

^1^ Graduate School of Education, Kyoto University, Kyoto, Kyoto, Japan

^2^ Wildlife Research Center, Kyoto University, Inuyama, Aichi, Japan

^*^ Corresponding author

E-mail: minami.toshiki.373@gmail.com

ORCID: 0000-0001-5476-3896

S1 Table. AIC results for the model selection in this study.

| 1. Identifying infantile facial features | | | | | | | | | |
| --- | --- | --- | --- | --- | --- | --- | --- | --- | --- |
|  | Null model | | | | Full model | | | | |
| EWFW | 1100.117 | | | | **1075.59** | | | | |
| FoLFaL | 906.9761 | | | | **724.5124** | | | | |
| EWFW | 1008.304 | | | | **858.3772** | | | | |
| NLHL | 869.6228 | | | | **788.3585** | | | | |
| NWFW | 888.7603 | | | | **839.387** | | | | |
| MWFW | 1160.364 | | | | **1137.672** | | | | |
| 1. Associations between infantile faces and caretaking behaviors | | | | | | | | | |
|  | Null model for Poisson | Poisson | | Zero-inflated Poisson | | Null model for negative binomial | Negative binomial | | Zero-inflated negative binomial |
| Affiliative contact duration with the mother (including all explanatory variables) | 33830.46 | 31396.95 | | 14656.86 | | 978.8119 | 983.8066 | | **915.8777** |
| Affiliative contact duration with the mother (after excluding the age variable) | 33830.46 | 31724.97 | | 14902.92 | | 978.8119 | 981.8849 | | **913.9064** |
| Affiliative contact duration with nonmothers (including all explanatory variables) | 18399.7 | 18080.63 | | 8475.734 | | 550.4012 | 555.9225 | | **541.5085** |
| Affiliative contact duration with nonmothers (before excluding the age variable) | 18399.7 | 18400.47 | | 8585.18 | | 550.4012 | 553.9227 | | **541.7889** |
| Affiliative contact duration with nonmothers (after excluding the age variable) | 18399.7 | 18111.02 | | 8672.398 | | 550.4012 | 552.3985 | | **540.4813** |
| 1. Development of infantile faces | | | | | | | | | |
|  | Null model | | Linear model | | Linear model with squared term | | | GAM | |
| Chonpe’01’21 | **-15.90611** | | -14.22361 | | -14.37325 | | | -14.97141 | |
| Cooper’90’01’21 | 2.11505 | | **-20.28548** | | -18.32175 | | | -20.28548 | |
| Kusha’98’21 | 0.5979557 | | **-22.40326** | | -21.14992 | | | -22.40326 | |
| Cooper’94’02’21 | -12.62248 | | -26.14362 | | -25.54728 | | | **-26.41706** | |
| Ai’02’21 | -8.419618 | | -19.53832 | | -19.3766 | | | **-24.09722** | |
| Cooper’14’21 | -7.988981 | | -23.87437 | | -24.45388 | | | **-26.28746** | |
| Cooper’94’01’21 | -7.558351 | | -7.731556 | | -18.53666 | | | **-20.47128** | |
| Cooper’89’02’21 | -19.39724 | | -21.90838 | | -24.08174 | | | **-24.57192** | |
| The numbers in bold are the lowest AIC among the comparing models. When some AICs were equal, simpler models were selected. | | | | | | | | | |
